# Supplementary material for: Tissue specific LRRK2 interactomes reveal a distinct striatal functional unit
Source: PLoS Comput Biol. 2023 Jan 30;19(1):e1010847. doi: 10.1371/journal.pcbi.1010847 (PMC9910798; doi:10.1371/journal.pcbi.1010847)
Supplement: S1 Fig — Fig A in S1 Fig. Overlap of LRRK2 interactors downloaded from PINOT, HIPPIE and MIST. Fig B in S1 Fig. Functions of LRRK2int A) The bar graph shows the numbers of interactors contributing to the enrichment of different functional groups. B) The 14 functional groups were then analysed via MCA to compare their composition in terms of LRRK2 interactors. Fig C in S1 Fig. Den_DEA1 presents the hierarchical clustering of tissues in terms of the overall expression pattern of the LRRK2int. Fig D in S1 Fig. Den_DEA2 presents the hierarchical clustering of LRRK2 interactors based on the expression patterns across different tissues (for the list of gene names refer to S10 Table). Fig E in S1 Fig. Co-expression coefficients distribution in 15 tissues. each tissue, the distribution of co-expression coefficients between LRRK2 and its interactors (in red) was compared to the “reference distribution” (co-expression coefficients between LRRK2 and 1000 randomly picked gene sets, in black). A: amygdala-LRRK2int; B: anterior_cingulate_cortex-LRRK2int; C: caudate-LRRK2int; D: frontal_cortex-LRRK2int; E: hippocampus-LRRK2int; F: hypothalamus-LRRK2int; G: nucleus_accumbens-LRRK2int; H: putamen-LRRK2int; I: spinal_cord_c-1-LRRK2int; J: substantial_nigra-LRRK2int; K: cerebellum-LRRK2int; L: blood-LRRK2int; M: liver-LRRK2int; N: lung-LRRK2int; O: kidney_cortex-LRRK2int. Fig F in S1 Fig. Den_Co-ex1 presents the hierarchical clustering of tissues in terms of the co-expression pattern between LRRK2 and its interactors. Fig G in S1 Fig. Den_Co-ex2 presents the hierarchical clustering of LRRK2 interactors in terms of their overall co-expression behaviours with LRRK2 across tissues analysed (for the list of gene names refer to Table S11). Fig H in S1 Fig. Overlap of primary databases in PINOT, HIPPIE and MIST (PDF) [file pcbi.1010847.s001.pdf]

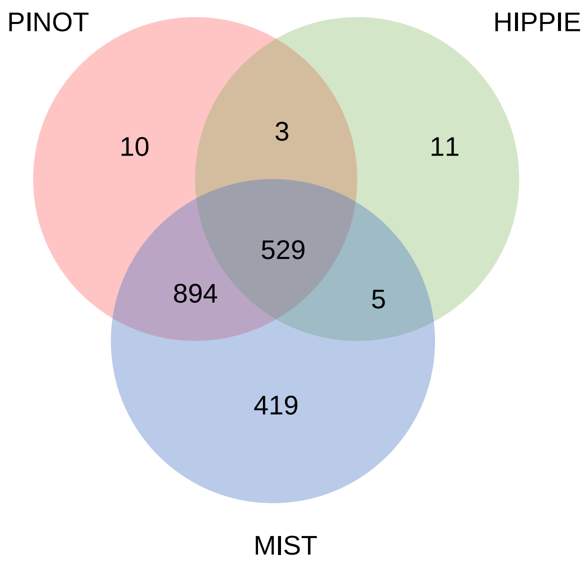

Fig A: Overlap of LRRK2 interactors downloaded from PINOT, HIPPIE and MIST

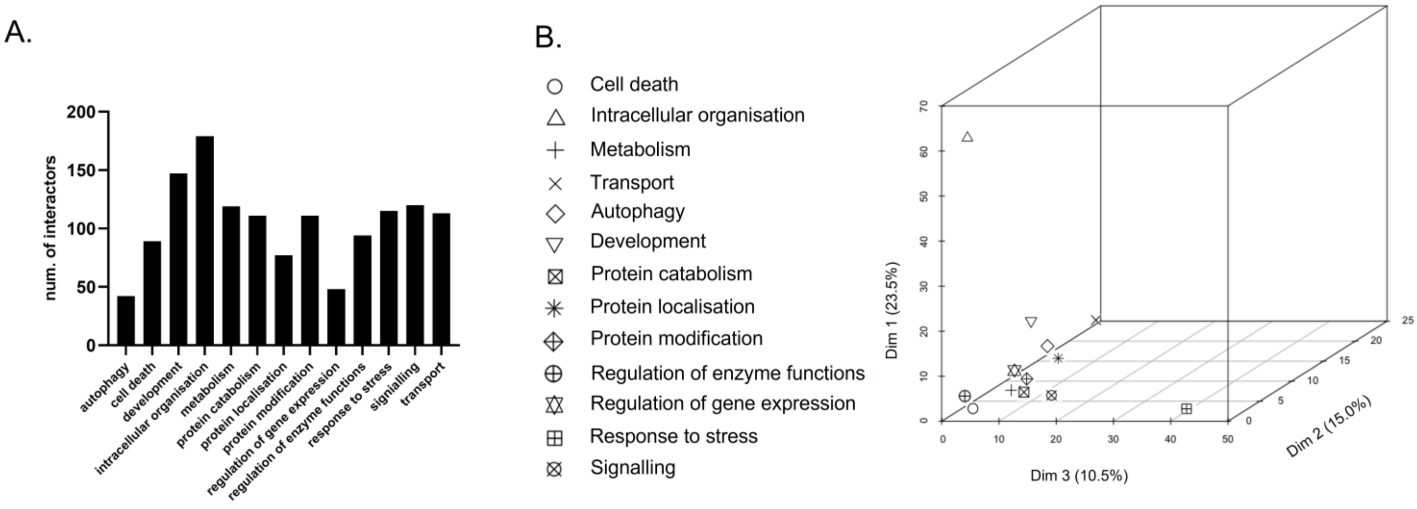

Fig B: Functions of LRRK2$_{int}$ A) The bar graph shows the numbers of interactors contributing to the enrichment of different functional groups. B) The 14 functional groups were then analysed via MCA to compare their composition in terms of LRRK2 interactors

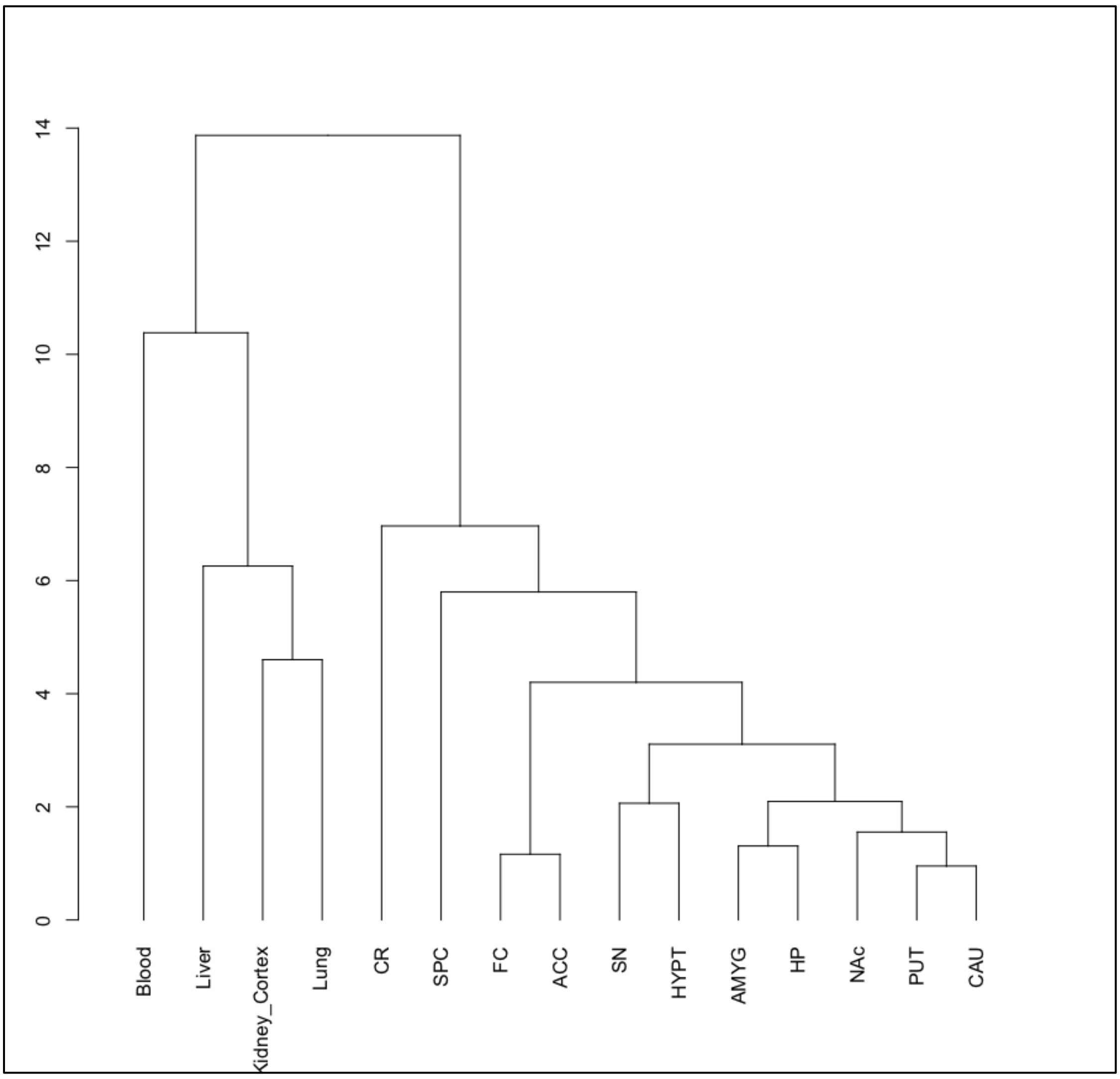

Fig C: Den_DEA1 presents the hierarchical clustering of tissues in terms of the overall expression pattern of the LRRK2int.

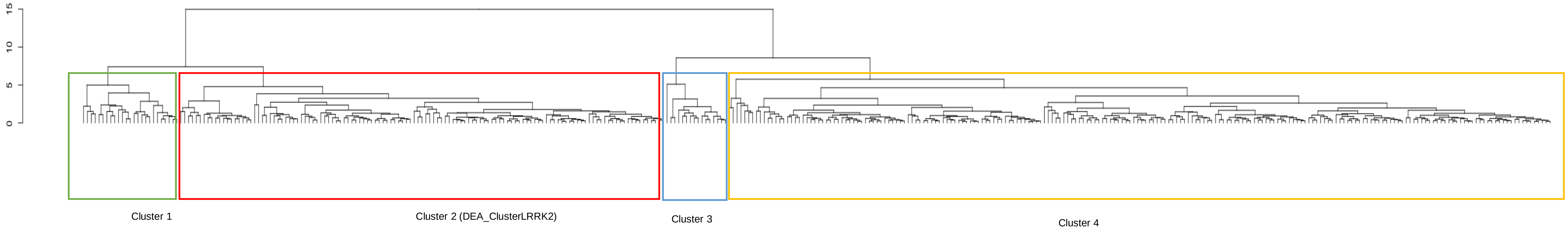

Fig D: Den_DEA2 presents the hierarchical clustering of LRRK2 interactors based on the expression patterns across different tissues (for the list of gene names refer to Table S10).

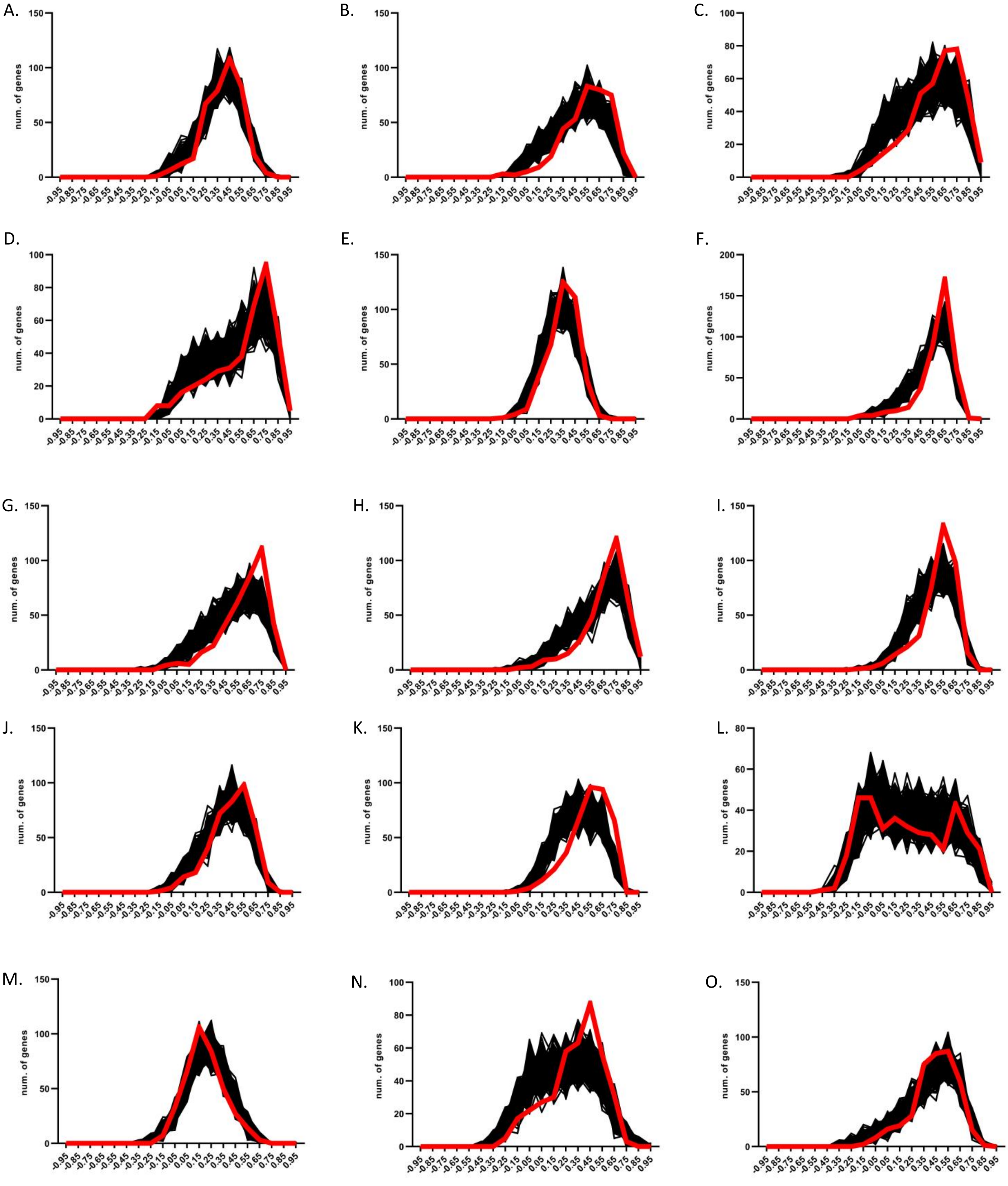

Fig E: Co-expression coefficients distribution in 15 tissues

each tissue, the distribution of co-expression coefficients between LRRK2 and its interactors (in red) was compared to the "reference distribution" (co-expression coefficients between LRRK2 and 1000 randomly picked gene sets, in black).

A: amygdala-LRRK2$_{int}$; B: anterior_cingulate_cortex-LRRK2$_{int}$; C: caudate-LRRK2$_{int}$; D: frontal_cortex-LRRK2$_{int}$; E: hippocampus-LRRK2$_{int}$; F: hypothalamus-LRRK2$_{int}$; G: nucleus_accumbens-LRRK2$_{int}$; H: putamen-LRRK2$_{int}$; I: spinal_cord_c-1-LRRK2$_{int}$; J: substantial_nigra-LRRK2$_{int}$; K: cerebellum-LRRK2$_{int}$; L: blood-LRRK2$_{int}$; M: liver-LRRK2$_{int}$; N: lung-LRRK2$_{int}$; O: kidney_cortex-LRRK2$_{int}$.

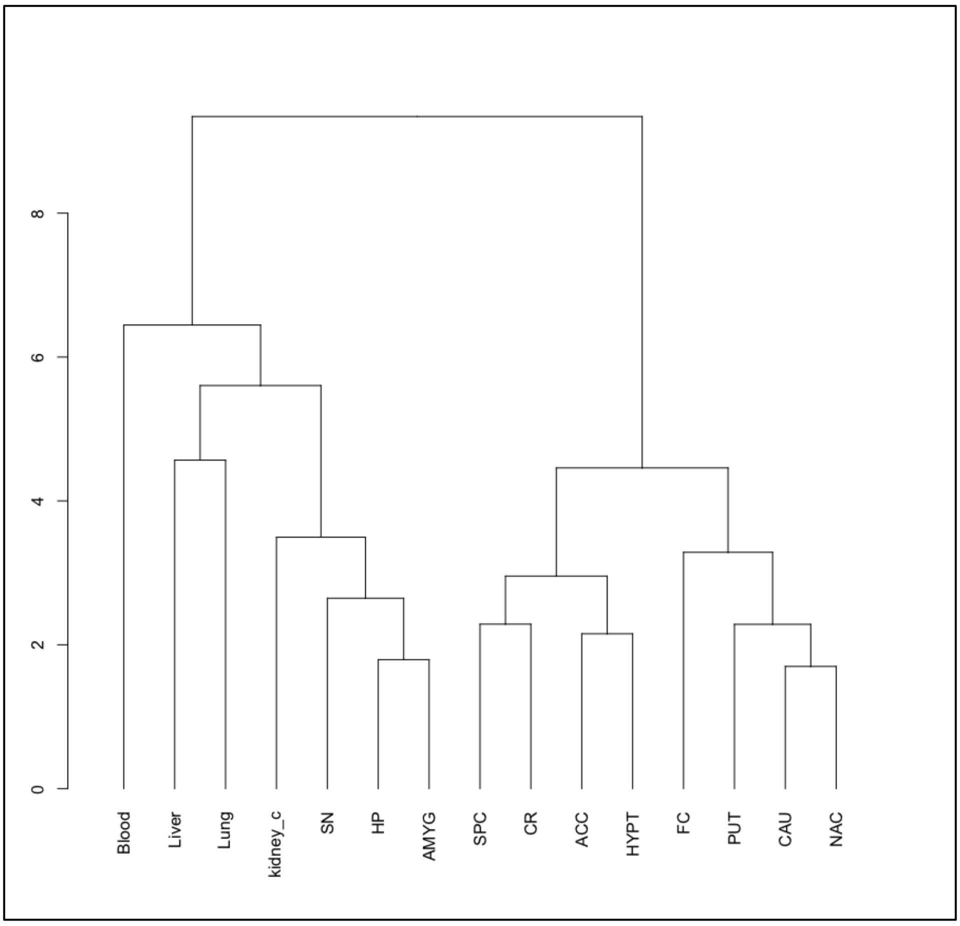

Fig F: Den_Co-ex1 presents the hierarchical clustering of tissues in terms of the co-expression pattern between LRRK2 and its interactors

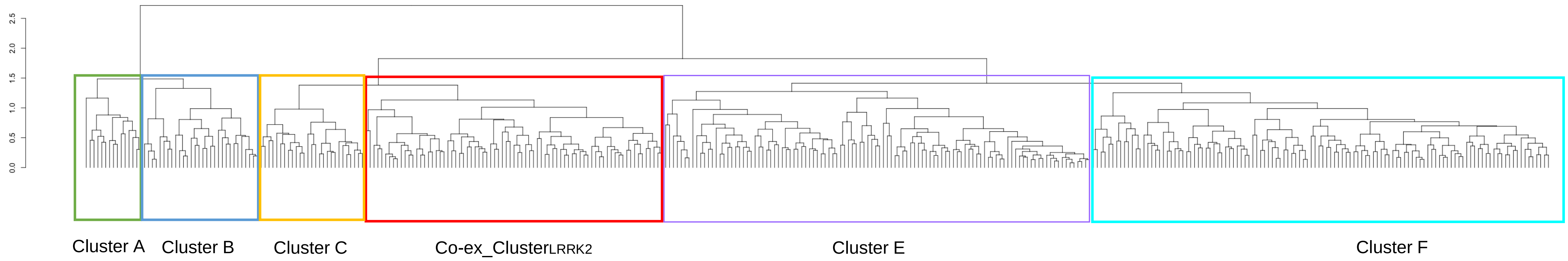

Fig G: Den_Co-ex2 presents the hierarchical clustering of LRRK2 interactors in terms of their overall co-expression behaviours with LRRK2 across tissues analysed (for the list of gene names refer to Table S11).

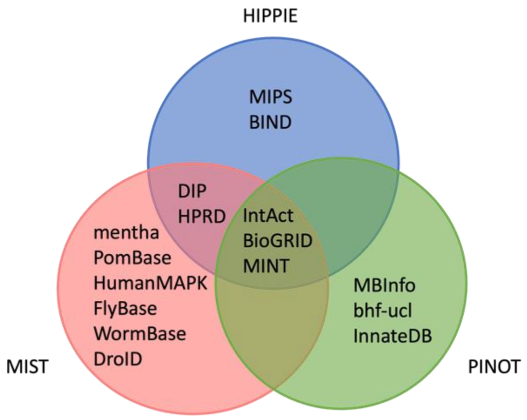

Fig H: Overlap of primary databases in PINOT, HIPPIE and MIST
