# Supplementary material for: Tissue specific LRRK2 interactomes reveal a distinct striatal functional unit
Source: PLoS Comput Biol. 2023 Jan 30;19(1):e1010847. doi: 10.1371/journal.pcbi.1010847 (PMC9910798; doi:10.1371/journal.pcbi.1010847)
Supplement: Attachment — Submitted filename: Response_to_Reviewers-FINAL_rm.UBC.docx [file pcbi.1010847.s003.docx]

**Manuscript: "Tissue specific LRRK2 interactomes reveal a distinct functional unit within the striatum"**

**Response to Reviewers**

Dear Editor, Dr. Andrade-Navarro,

Thank you for the opportunity to submit a revised version of our manuscript "*Tissue specific LRRK2 interactomes reveal a distinct functional unit within the striatum*" for publication in *PLOS Computational Biology*. We appreciate the time and effort you and the reviewers dedicated to the evaluation of our manuscript and are grateful for the insightful comments. We replied to all the points raised by the reviewers and have incorporated most of the suggestions within the revised version of the manuscript.

In addition, we have simplified the figures and re-run the analyses to account for the removal of the protein UBC from the LRRK2 interactome.

Changes are highlighted with track-changes within the revised manuscript.

Please see below for a point-by-point response to the reviewers’ comments.

We think that our revisions have improved the manuscript and we hope you and the reviewers will find this updated version suitable for publication.

With many thanks and kindest regards

Claudia Manzoni

__________________________________________________________________________

**Reviewers’ Comments to the Authors:**

**Reviewer 1**

*The authors construct brain-specific interactomes around the Parkinson's disease protein LRRK2. To this end, they combine PPI data with gene expression information from different public resources. Their work is interesting yet mostly describing the different generated networks. They highlight a few interesting observations that could contribute to / explain characteristics of LRRK2 pathology. Eg. they observe similarities in different subregions of the striatum. Overall, the work is clear and does not contain any major flaws.*

*Major comment 1:*

*Are the results of the MCA shown in Figure 3B specific to LRRK2 interactors? Or is the clustering a more general property of those functional categories? Eg do protein catabolism and protein modification cluster also when all proteins annotated with those categories are considered? Or only for the subset of LRRK2 interactors?*

**Response:**

We thank the reviewer for this comment that has made us re-think our approach.

In order to reply to this specific question, we followed 2 different strategies:

1. We considered all the genes annotate in Gene Ontology for the GO-BPs that in our analysis were grouped into the 13 significant functional blocks. We performed MCA on this set of data that we refer to as “**General-Set**”. This analysis is presented below in panel A. In our understanding, this is exactly the type of approach suggested by the reviewer: *“Do protein catabolism and protein modification cluster also when all proteins annotated with those categories are considered?”*
2. We considered all the significant functional blocks derived from the enrichment of the interactome of another protein, different from LRRK2. We selected NFKB1. We performed MCA on this set of data that we refer to as “**NFKB1-Set**”. This analysis is presented below in panel B.

The results from 1 and 2 were then compared to the MCA results obtained for the **LRRK2-set** presented in our manuscript.

***Figure 1.*** ***Panel A)*** *MCA results for the General-Set enrichment;* ***Panel B)*** *MCA results for the NFKB1-Set enrichment. A total of 561 interactors for NFKB1 (UniProtKB: P19838) were derived from PINOT (NFKB1_int_). GO-BP enrichment analyses were performed with the same settings via g:Profiler as described in the manuscript. The GO-BP terms were then filtered with the same thresholds set for the enrichment of LRRK2_int_ (i.e., term size ≤ 1500 & keep only terms with NFKB1 involved). Terms were finally allocated into functional categories based on the same dictionary used for LRRK2-Set and General-Set.*

The General-Set shows similar clustering results when compared to the LRRK2-Set. For example, in both sets, “protein modification”, “protein catabolism” and “metabolism” showed in the same cluster. This suggests that the list of proteins contributing to these functional blocks have a very large overlap in general. In other words, given the annotations we have at the moment in Gene Ontology, the proteins contributing to “protein catabolism” are, for the most part, also contributing to “protein modification” and “metabolism”. These 3 functional blocks are linked together irrespective of LRRK2.

However, the General-Set shows also different clustering results when compared to the LRRK2-Set. The functional block of “regulation of gene expression” was clustered with “protein modification”, “protein catabolism” and “metabolism” in the LRRK2-Set. This is potentially a LRRK2-specific finding as it does not happen when we consider the entire set of proteins annotated in Gene Ontology.

When we looked at the interactome of NFKB1, the functional blocks of: “protein modification” and “protein catabolism” were not significantly enriched, thus no comparison was performed. Moreover, “regulation of gene expression” and “metabolism” were not clustered. This is an interesting observation, in fact, the NFKB1 interactome contains a comparable number of interactors to the LRRK2 interactors: 561 NFKB1 interactors vs 407 LRRK2 interactors. The functional enrichment showed many differences suggesting the results of the pipeline we implemented within this paper are specific.

**In summary,** we agree with the reviewer that part of the clustering (i.e. “protein modification” and “protein catabolism”) might be driven by the way the ontologies are composed in Gene Ontology; however, part of the clustering (i.e. “protein localization”) appears to be specific to the LRRK2 scenario.

However, we agree with the reviewer that the clustering results are highly biased by the ontology and how it is built. Therefore, in the main manuscript, we toned down the discussion around the clustering results. We also considered that the 3 first components in MCA only explain 49% of the data variability and thus it is probably advisable not to draw any conclusions.

We have therefore changed the results section with the following (page 11):

*“With the exception of ”metabolism”, “regulation of gene expression”, “protein modification” and “protein catabolism” that are very close in the MCA space (this might be due to similarity in the ontology behind these terms); no other cluster was confidently identified. This suggested the biological processes associate with LRRK2 and represented by the different functional blocks are probably sustained by distinct sets of LRRK2 interactors.”*

In fact, we feel that it is not the “clustering” to be of interest here; but the “non-clustering” properties that are important. We think it is important to highlight that the different functional groups don’t cluster all together in a single/unique cluster, meaning they should be associated to different sets of LRRK2 interactors. This observation is relevant for supporting the hypothesis of tissue-specific LRRK2 functions as stated in the discussion:

*“This result confirmed that i) the LRRK2_int_ is indeed involved in a wide range of different functions and ii) when looking at each of these functions, it appears that they are driven by distinct sets of LRRK2 interactors. In other words, LRRK2 interactors do not contribute, as a group, to the enrichment of all the LRRK2 associated functions. Rather, specific sets of LRRK2 interactors contribute to the enrichment of specific LRRK2 associated functions. This note is of importance as it sustains the hypothesis that differential expression of groups of LRRK2 interactors in different tissues might indeed be responsible for the specialisation of LRRK2 functions.”*

*Major comment 2:*

*Which version of the different resources did the authors use? Eg. the current version of HIPPIE lists 1,543 interactions formed by LRRK2, so I suppose the authors used a previous version. They should indicate for all databases which version they used to allow for reproducibility of the results.*

**Response:**

The reviewer is correct, we had indicated the date of the analysis but not the version of the tools used. These are: HIPPIE v2.2, PINOT v1.1 and MIST v5.0. We have now added this information (Page 3/4):

*“PINOT (v1.1) (http://www.reading.ac.uk/bioinf/PINOT/PINOT_form.html) (Tomkins et al, 2020), HIPPIE (v2.2) (http://cbdm-01.zdv.uni-mainz.de/~mschaefer/hippie/index.php) (Alanis-Lobato et al, 2017) and MIST (v5.0) (https://fgrtools.hms.harvard.edu/MIST/) (Hu et al, 2018) were queried to download “homo sapiens” PPIs for LRRK2 (UniProt ID: Q5S007, 21 October 2020)”.*

*Major comment 3:*

*To which degree are the functional enrichments a consequence of technical and research biases? I understand from the description in the Methods section that no background was used for determining the functional enrichments. However, the set of interacting proteins is already biased towards certain protein classes (eg highly expressed) due to mostly technical biases. Filtering towards interaction partners detected several times as the authors do, will likely further bias the network towards well studied proteins. The authors should test to which degree the GO term analysis results are due to those biases by testing against a suitable background (at least all proteins in the PPI networks used but even better comparing to proteins with similar network topological properties).*

**Author response:**

Thank you for this comment, this is an interesting observation and touches upon one of the limitations of protein network analysis which is ascertainment bias. We agree with the reviewer that our LRRK2 interactome is potentially biased toward well-studied proteins. However, this bias is intrinsic of PPI network analysis because this analysis is based on the use of data that is incomplete by definition. We addressed this point in the discussion, but following the reviewer comments we decided to stress this more.

We have therefore added the following paragraph:

*“It is important to consider, however, that PPI analyses suffer from ascertainment bias as more studied and more abundant proteins tend to be more represented. Additionally, there is no tissue/cell specific dimension to the PPI data as protein interactions from experimental data tend to be generated in model systems (i.e. HEK293 cells) or completely in vitro systems (proteo-arrays).”*

We also agree that the quality control (QC) pipeline (i.e., keep only the interactions with a final score ≥ 3) has the potential of pushing the LRRK2 interactome towards well-studied proteins. However:

1. we believe this QC process is necessary to ensure the reliability of the whole interactome preventing it from being affected by false positives brought in by high-throughput techniques (e.g protein array and MS results represent ~22.5% of the “interaction detection methods” for the LRRK2 interactions we downloaded, and often [~11%] they are not confirmed by other methods).
2. the filter used (final score ≥ 3) is not very stringent. In fact, an interactor receives a final score of 3 if it has been identified i) in 2 different publications with the same technique or ii) in 1 single publication with 2 different techniques.

In summary, we believe the filter used at QC is not strong enough to relevantly increase the bias toward well-known proteins; and the benefit of using the filter to remove false positives (principally derived by non-replicated high through-put screening results) outweighs the bias it might introduce.

In terms of “background for GO enrichment analysis”, we used the default setting of g:GOSt which provides enrichment against “only annotated genes”. This specific background allows g:GOSt to calculate enrichment by comparing the frequency of each single GO:BP term in the set of proteins constituting the LRRK2 interactome vs all genes in the human genome with at least one annotation in Gene Ontology. Genes that lack annotations (because they are less studied genes/proteins) are omitted from statistics. Such background is suggested in the default settings to reduce the number of significant results (<https://biit.cs.ut.ee/gprofiler/page/docs#statistical_domain_scope>).

We have now specified this better in materials and methods at page 4.

*“The general LRRK2_int_ was analysed by Functional Enrichment Analysis using the tool g:GOSt on 8 Nov 2022 (g:Profiler (*[*https://biit.cs.ut.ee/gprofiler/gost*](https://biit.cs.ut.ee/gprofiler/gost)*) (Raudvere et al, 2019). The parameters were set as follows: organism - Homo sapiens (Human); data source - GO biological process (GO-BPs) only; statistical domain scope (i.e. background) – annotated genes only; statistical method - Fisher’s one-tailed test; significance threshold – Bonferroni correction (threshold = 0.05).”*

In order to examine how background settings affect the enrichment results, as suggested by the reviewer, we conducted another GO-BP enrichment analysis on the same set of LRRK2 interactors (n = 407) changing the background to “all known genes” (i.e, all genes of *homo sapiens* in the Ensembl database). A total of 1195 GO-BP terms with p-values < 0.05 were returned from g:GOSt with the background set as “All known genes” (Set 1), as compared to 480 significant terms found with the background of “Only annotated genes” (Set 2). We then allocated the GO-BPs retrieved from Set 1 into functional blocks using the same dictionary as described in the manuscript. We compared the contribution of GO-BPs in each functional block towards the whole enrichment between Set 1 and Set 2 via Two-sample Z-test (please see table below, p-values were adjusted via Bonferroni’s test).

The result shows that out of 14 functional blocks, only 4 of them showed statistically significant differences. Set 1 contained significantly more terms related to “protein localisation” (3.8% vs. 0, adjusted-p < 0.0001), “development” (13.0% vs. 7.9%, adjusted-p = 0.0285), immune system (5.2% vs. 0.4%, adjusted-p < 0.0001) and “regulation of gene expression” (7.9% vs. 3.8%) than Set 2. No significant difference was found in other functional blocks. Therefore, we conclude that background settings are not greatly impacting our functional analysis for the LRRK2 interactome as only 4 categories out of 14 are affected by the background change.

| block | freq.all (Set 1) | freq.annotated (Set 2) | adj.p |
| --- | --- | --- | --- |
| autophagy | 21 (1.8%) | 14 (2.9%) | 1 |
| cell cycle | 40 (3.3%) | 10 (2.1%) | 1 |
| cell death | 62 (5.2%) | 31 (6.5%) | 1 |
| development | 155 (13.0%) | 38 (7.9%) | 0.0285 |
| immune system | 62 (5.2%) | 2 (0.4%) | <0.0001 |
| intracellular organisation | 117 (9.8%) | 61 (12.7%) | 1 |
| metabolism | 126 (10.5%) | 61 (12.7%) | 1 |
| protein catabolism | 36 (3.0%) | 32 (6.7%) | 1 |
| protein localisation | 45 (3.8%) | 0 | <0.0001 |
| protein modification | 46 (3.8%) | 23 (4.8%) | 1 |
| regulation of enzyme functions | 36 (3.0%) | 24 (5.0%) | 1 |
| regulation of gene expression | 94 (7.9%) | 18 (3.8%) | 0.0255 |
| signalling | 148 (12.4%) | 62 (12.9%) | 1 |
| transport | 109 (9.1%) | 46 (9.6%) | 1 |

It is also interesting to note that functional enrichment performed on a different protein interactome of similar topological properties (LRRK2 interactome vs NFKB1 interactome, please refer to the reply to major point 1) resulted in a very different set on enriched terms.

*Minor comment 1:*

*Is LRRK2-ints the same us LRRK2_ints_ (where _ denotes subscript)? In general abbreviations should be clearly defined at their first occurrence in the manuscript and then consistently used.*

**Response:**

Thank you very much for highlighting this typo and apologise for the confusion. Yes, they both refer to “LRRK2 interactomes”. We have now double-checked the entire manuscript to make sure the abbreviation “**LRRK2_ints_**” is used consistently (you will notice corrections in track-changes).

*Minor Comment 2:*

*Partly the organization of information into Methods and Results could be done better. Eg both the Results as well the Methods section have a section entitled "**RNA-Seq data download and quality control" with highly redundant content. The manuscript could substantially benefit from avoiding this redundancy (in most cases I'd shorten the Results part).*

**Response:**

We think the reviewer is correct in pointing this out; we have reorganised the Result chapter by taking this comment into consideration and also major comment 1 from reviewer #2. In summary, we have changed the titles making sure there is no overlap between results and methods, removed redundancies with the methods (in particular for the expression analyses), simplified the results, modified the end part of the results, removed 1 result chapter (and associated method section) and 1 figure.

The extent of these changes is too large to be reported here, thus we refer the reviewer to the new “Results” chapter in the manuscript.

The methods sections now are titled as follows:

- Protein-protein interaction (PPI) download
- Functional characterisation of the general LRRK2_int_ enrichment
- RNA-Seq data download and quality control
- Differential expression analysis
- Co-expression analysis

The results sections now are titled as follows:

- Construction of the general LRRK2_int_
- Functional enrichment analysis of the general LRRK2_int_
- Differential expression analysis of the general LRRK2_int_
- Co-expression analysis of the general LRRK2_int_
- Tissue-specific LRRK2 interactomes
- Differentiating LRRK2:Rab interactions in the CNS and the periphery

*Minor Comment 3:*

*"These tools extract PPIs from multiple primary, manually curated databases allowing to effectively text mine the available PPI literature” -> the term "text mining" is misleading here*

**Response:**

We completely agree with the reviewer. The have modified the text as follows (Page 17):

*From:*

*“These tools extract PPIs from multiple primary, manually curated databases allowing to effectively text mine the available PPI literature”*

To:

*“These tools extract PPIs from multiple primary, manually curated databases allowing to effectively retrieve the available PPI literature”*

*Minor Comment 4:*

*I find the first paragraph of the Discussion misleading: why discussing factors that distinguish the three PPI source databases used (MIST, HIPPIE and PINOT) but were not really taken into account or are relevant for the study (eg the scoring of PPIs or the download time of interactions).*

**Response:**

Thank you for this comment, we think the reviewer is right; we discuss some differences across tools that are not relevant for the study, thus they are unnecessary in our discussion.

We have therefore removed this comparison from the discussion. We decided to focus only on explaining how a combination of the 3 PPI databases (MIST, HIPPIE and PINOT) results in a more comprehensive interactome of LRRK2. We have also added a supplementary figure to help us simplifying the text.

The revised discussion reads as follows (page 17/18):

*“**These tools extract PPIs from multiple primary, manually curated databases (Figure S6) allowing to effectively retrieve the available PPI literature. We removed all scoring filters while downloading interactions. This allowed us to retrieve as many LRRK2 interactions as possible from the literature. Therefore, to the best of our knowledge, the interactome presented in this manuscript represents the most comprehensive reconstruction of the LRRK2 interactome to date.”*

**Reviewer 2**

*By integrating data from different public databases, Zhao and colleagues provide a comprehensive and technically sound computational analysis of the tissue specificity of the LRRK2 interactome. In this work, the authors overlaid tissue specific expression with protein-protein interaction (PPI) data available in the databases. Data confidence scores for individual LRRK2 interactors have also been considered. The tissue specificity of the LRRK2 associated signaling has long been discussed in the field, especially in the context of organ specific phenotypes. For this reason, the present bioinformatic analysis may definitely contribute to a better understanding of LRRK2 signaling by systematically annotating the tissue specific expression of individual LRRK2 PPIs. This is also of particular interest for the development of specific and effective targeting strategies as it may help to understand and (even) reduce tissue specific (on-target) side effects of LRRK2-centric intervention in the future.*

*Major Comment 1:*

*While the manuscript is very much focusing on technical details, the biological meaning of the results remains largely unclear. I would therefore like to encourage the authors to provide a more detailed discussion on the functional outcome of the study to help the reader to better understand the tissue specific LRRK2 function and to potentially come up with testable hypotheses.*

**Response:**

We thank the reviewer for this comment as we believe this is a very important point. With this manuscript we intend to present the LRRK2 model/interactome we generated as a tool to be used by wet-lab researchers to identify testable hypotheses and support their experimental research. This reviewer’s comment (and also minor point 2 from reviewer#1 highlighting the complexity of the manuscript) made us realize we did not achieve this goal with the previous version of the manuscript.

We have therefore worked on the manuscript to convey in a better and simpler form the biological meaning/application of the LRRK2 interactome we built. For this reason, we have completely modified the final part of the results, removed 2 figures (6 and 7), produced a new figure 6 and changed the associated supplementary tables.

The extent of these changes is too large to be reported here, thus we refer the reviewer to the new “Results” chapter in the manuscript.

In summary: after introducing how the general LRRK2_int_ has been built and the calculations for the differential expression and co-expression of the LRRK2 interactors (with associated clustering results highlighting the importance of the striatum cluster), we decided to introduce an example of how the LRRK2 interactome + the expression analyses can be used to generate tissues specific LRRK2 interactomes.

First, we introduced a new Supplementary Table 12 that is the tool that readers can download to generate their own tissue-specific LRRK2 interactomes. Of note, this does not require any coding, the table can be easily filtered just by setting 2 thresholds: 1 for the DEA analysis and 1 for the co-expression analysis.

- The first example of use for Supplementary Table 12 is reported in the new results chapter titled: “Tissue specific LRRK2 interactomes”. We have selected 4 brain regions based on the clustering results (putamen, caudate, nucleus accumbens and cerebellum) to exemplify how to generate their tissue-specific LRRK2 interactomes using Supplementary Table 12. We have set 2 arbitrary (but stringent) thresholds, extracted the top LRRK2 interactors expressed in these 4 brain regions and compared them following functional enrichment, showing that there are divergent LRRK2_int_ functions between the striatum (putamen, caudate, nucleus accumbens) and the cerebellum. These results are reported in the new Figure 5 (reproduced here below) and led us to hypothesise that the LRRK2 interactome (thus probably LRRK2 itself) is involved in developmental processes related to a specific structure of the cytoskeleton (lamellipodium) in the striatum vs response to oxidative stress and transport of mitochondria over the cytoskeleton in the cerebellum.
- The second example of use for Supplementary Table 12 is reported in the result chapter titled “Differentiating LRRK2:Rab interactions in the CNS and the periphery”. This chapter was already present in the previous version of the manuscript and we have left it mainly unchanged (with the exception of some revisions to simplify it).

Finally, we have also added a small analysis to show the functional annotation of the 124 and 77 proteins involved in the DEA and the co-exp clusters. These new results are reported in Tables 1 and 2.

*Major Comment 2:*

*If I understand it correctly, the major approach is to overlay tissue specific expression profiles with the published LRRK2 interactome. However, the experimental data might have a bias due to the over representation of some techniques and cell systems which also impacts the confidence score of individual interactors. I would appreciate if the authors could comment on how the experimental bias is considered/compensated in the study.*

**Response:**

Thank you for this comment, this is an interesting observation and touches upon one of the limitations of protein network analysis which is ascertainment bias. In particular the reviewer mentioned problems associated with: 1) over-represented techniques and 2) over-represented cell systems.

**1. Over-represented techniques** – we completely agree there are over-represented techniques. In our opinion, this happens because some techniques that are very similar are indicated as different techniques, with different method codes within the method ontology. This has the potential of impacting on the calculation of the individual confidence scores as the reviewer is correctly pointing out. For example: “Two hybrid fragment pooling approach MI:0399” and “Two hybrid bait and prey pooling approach MI:1113” are catalogued as different techniques, but they are very similar approaches. If we were to follow the method classification from the methods ontology, a protein interaction identified by both these 2 techniques would receive an inflated confidence score, like if these were 2 independent/different experimental approaches. We agree with the reviewer that this is potentially an experimental bias. Thus, we have compensated this bias in our analysis by implementing the PINOT scoring method that combines similar detection methods into categories (e.g. “Two hybrid fragment pooling approach MI:0399” and “Two hybrid bait and prey pooling approach MI:1113” are allocated in the same category: “Two Hybrid”). Therefore, the method score (MS) is calculated as the number of different categories of detection methods used to detect a PPI, instead of the number of different detection methods used to detect a PPI. In this way, the bias due to the over-represented detection methods is minimised.

To show how our scoring method helps in reducing the bias of over-represented techniques, we compared the distribution of MS for LRRK2 interactors downloaded from HIPPIE without/with categorising similar detection methods (MS1 vs. MS2, please see graph below).

The graph shows that without method clustering (MS1), LRRK2 interactors gained higher scores due to the bias caused by over-represented techniques. However, after allocating similar methods into categories (MS2) the distribution of scores was significantly reduced (t-test p-value < 0.001). This suggests that our quality control process can significantly reduce the bias from replicated detection methods.

**2. Over-represented cell systems** – It is very difficult to identify the cell system in which a PPI has been identified as this information is not currently provided by HIPPIE, PINOT and MIST, nor by the primary databases using the standard PSI-MI TAB 2.5 annotation file. Thus, it was impossible to mitigate this bias in our analysis.

However, we went back to Intact, one of the PPI primary databases used by HIPPIE, PINOT and MIST to retrieve the LRRK2 interactions using the PSI-MI **TAB 2.6** annotation file as this is the version that reports the “host organism” indication. We evaluated the distribution of the host organisms used to identify the LRRK2 interactors. Results are reported in the table below and it is quite striking that the majority of the interactions have been reported from studies in HEK293 cell lines.

Since we don’t have access to the PSI-MI **TAB 2.6** annotation files from other primary databases (particularly Biogrid which is the second largest database used by MIST, HIPPIE and PINOT) we cannot be sure this distribution is replicated for all the LRRK2 annotations. However, we can infer this to be the case. We have therefore added a sentence to our discussion to raise the attention of the reader to this potential bias of the data.

*“It is important to consider, however, that PPI analyses suffer from ascertainment bias as more studied and more abundant proteins tends to be more represented. Additionally, there is no tissue/cell specific dimension to the PPI data as protein interactions from experimental data tend to be generated in model systems (i.e. HEK293 cells) or completely in vitro systems (proteo-arrays).”*

*Major Comment 3:*

*As the readability of the figures is limited in several cases, I would recommend increasing the font size for the annotations. In particular, the informative value of Figure 6 is quite limited due to unreadable protein/ gene names.*

**Response:**

We have increased the font size for annotations in our graphs as suggested by the reviewer. As for Figure 6 (now Figure 5), a simple increase in font size was not sufficient to improve readability; therefore, we re-organised it, please refer to major comment 1.
